# Coupling Ultrafiltration-Based Processes to Concentrate Phenolic Compounds from Aqueous Goji Berry Extracts

**DOI:** 10.3390/molecules25163761

**Published:** 2020-08-18

**Authors:** Carmela Conidi, Enrico Drioli, Alfredo Cassano

**Affiliations:** 1 Institute on Membrane Technology, ITM-CNR, Via P. Bucci 17/C, 87036 CS Rende, Italy; e.drioli@itm.cnr.it; 2 Department of Engineering and of the Environment, University of Calabria, Via P. Bucci 45/A, 87036 CS Rende, Italy; 3 State Key Laboratory of Materials-Oriented Chemical Engineering, College of Chemical Engineering, Nanjing Tech University, Nanjing 210009, China; 4 Center of Excellence in Desalination Technology, King Abdulaziz University (KAU-CEDT), Jeddah 21589, Saudi Arabia

**Keywords:** *Lycium barbarum* L., Goji berries, ultrafiltration, antioxidants, phenolic compounds, membrane processes

## Abstract

In this work, a membrane-based process for the purification and concentration of antioxidant compounds from aqueous Goji (*Lycium barbarum* L.) berry extracts was investigated. The aqueous extract was previously clarified with hollow fiber ultrafiltration (UF) membranes in order to remove suspended solids and β-carotene and to produce a clarified extract enriched in phenolic compounds. Then, three UF flat sheet polyamide membranes with a molecular weight cut-off (MWCO) in the range 1000–3500 Da were tested to purify and concentrate phenolic compounds from the clarified extract. The effect of MWCO and transmembrane pressure (TMP) on the performance of selected membranes in terms of productivity and selectivity towards total dissolved solids (TDS), total phenolic compounds (TPC), total carbohydrates (TC) and total antioxidant activity (TAA) was evaluated. Experimental results indicated that the 2500 Da membrane exhibited a lower fouling index, higher cleaning efficiency, lower rejection towards carbohydrates (lower than 30%) and higher rejection towards phenolic compounds (higher than 50%) in comparison to the other investigated membranes. The inclusion of a diafiltration process in the treatment of the clarified extract with this membrane in a spiral-wound configuration improved the concentration of sugar compounds in the permeate stream and increased the purification of phenolic compounds in the retentate fraction.

## 1. Introduction

Goji (*Lycium barbarum* L.) is a traditional Chinese medicinal plant, specifically a Solanaceous shrub, whose fruits, well known as Goji berries, are widely consumed in fresh, dried or juice form as well as in combination with tea, wine, meat and vegetarian meals [1,2].

Goji berries were introduced in Europe and North America at the beginning of the 21th century and their consumption has increased rapidly due to their claimed nutritional and beneficial properties [3]. In particular, Goji berries, advertised as “superfood” in Western Countries, are widely consumed as dried fruits or as concentrated extracts, as well as transformed into functional products or dietary supplements with different formulations (juices, yogurt products, cake, medicinal foods and cosmetics) [1,4].

Epidemiological studies have suggested that Goji berries have a wide range of biological properties including antioxidant, anticarcinogenic, immune enhancing and cardiovascular protective properties as well as neuroprotection, and hypoglycaemic and anti-aging effects [5]. Furthermore, in Traditional Chinese Medicine, *Lycium barbarum* can treat various diseases related to the liver, immune system, infertility, circulation, fatigue, dizziness, and headache [6,7]. The health benefits of Goji berries have been attributed to their chemical composition, which includes different classes and types of antioxidants such as polyphenols and carotenoids, as well as polysaccharides, salts, vitamins, and other micronutrients [4,8,9,10]. A perusal of the literature revealed that the main polyphenols identified in Goji berries are phenolic acids and flavonoids including caffeic acid, chlorogenic acid, *p*-coumaric acid, kaempferol-3-*O*-rutinoside, rutin and quercetin-diglucoside. These compounds are well known as radical scavengers and antioxidants capable of preventing cancer, cardiovascular disease, and other chronic diseases [11]. Goji berries are also an excellent source of different classes of carotenoids, which are responsible for their characteristic bright orange and red colors [12]. Among the carotenoids identified in Goji berries, zeaxanthin, an isomer of lutein and a derivative of β-carotene, has been proven to prevent chronic diseases such as age-related macular degeneration induced by excessive sun exposure and other oxidative processes [13,14]. In addition, carotenoids are used as non-toxic and natural colorants in the food industry and possess various biological activities mainly related to their antioxidant potential [8].

In view of the impact of carotenoids and polyphenols on human health and of the growing interest and demand for functional foods and pharmaceuticals derived from these compounds, there is an urgent need to develop effective and selective methods for the extraction, purification and separation of these bioactive natural products.

Several methods have been developed to improve the isolation and the production of bioactive compounds from natural sources, which include various extraction techniques and metabolic engineering [15]. Solvent extraction is traditionally used to obtain specific compounds from plants thanks to its low processing cost and ease of operation. However, this process uses toxic solvents, and requires an evaporation/concentration step for recovery, as well as large amounts of solvent and expensive disposal procedures [16].

Innovative extraction techniques have been proposed to replace common solvent extraction. They include pressurized liquid extraction [17], sub and supercritical extraction [18], ultrasound-assisted extraction [19], microwave-assisted extraction [20] and membrane processing [21]. In particular, pressure-driven membrane processes such as microfiltration (MF), ultrafiltration (UF), nanofiltration (NF) and reverse osmosis (RO), as well as integrated systems, have been increasingly studied over the last few years for the extraction, purification, fractionation and concentration of biologically active compounds from different agro-food products and by-products [22,23,24,25]. These processes offer several advantages over competitive methodologies since they can be operated in mild operating temperatures, thus favoring the preservation of nutrients and flavor components; additional advantages include small area-requirement, no use of chemical additives, high separation efficiency and easy scale-up.

Depending on the pore diameter, chemical nature of the membranes, and the operating conditions, it is possible to concentrate and fractionate the different bioactive compounds present in vegetables and food extracts, including sugars, polyphenols and carotenoids. In order to reach the maximum purification process, diafiltration conditions can be employed, using water as a solvent. Diafiltration reduces the concentration of low molecular weight hydrosoluble compounds, such as sugars, while retaining less hydrosoluble compounds, macromolecular compounds and molecular complexes [26].

To our knowledge, the use of membrane technology for the extraction, purification and concentration of biologically active compounds (including β-carotene and polyphenols) from Goji berries has not yet been reported in the literature. The optimization of a membrane-based process for the recovery of these compounds from Goji berries offers a new perspective for the food, cosmetic and pharmaceutical industries to replace the common synthetic pharmaceuticals by natural nutraceuticals.

The aim of this work was to evaluate the potential of an integrated and environmentally friendly membrane process for the production of natural concentrated extracts enriched in carotenoids and polyphenols from Goji berries. The whole process is based on an aqueous extraction of Goji berries followed by clarification and fractionation/concentration processes with UF membranes of different molecular weight cut-off (MWCO) and polymeric material. The performance of selected membranes was evaluated in terms of productivity, membrane fouling and selectivity towards β-carotene, polyphenols and sugars. The UF process was also combined with diafiltration in order to increase the purification of some bioactive compounds from sugars.

## 2. Results and Discussion

### 2.1. Clarification of Aqueous Extract with Hollow Fiber Membranes

**Figure 1:**
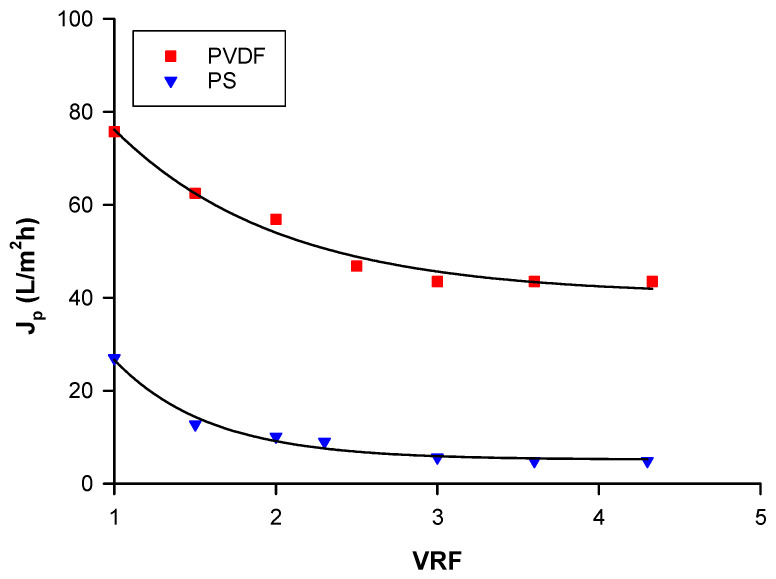
Ultrafiltration of Goji berry aqueous extracts with polyvinylidene fluoride (PVDF) and polysulphone (PS) membranes. Permeate flux as a function of volume reduction factor (VRF). Operating conditions: transmembrane pressure (TMP), 0.8 bar; feed flowrate (Q_f_), 280 L/h; temperature (T), 21 ± 1 °C.

Permeate flux is an important parameter in membrane systems because it is related to the productivity of the process. Figure 1 shows the behavior of permeate flux as a function of volume reduction factor (VRF) in the clarification of the aqueous extract with follow fiber (HF) membranes in the selected operating conditions. A typical trend was observed, characterized by a rapid initial decline of the permeate flux until a VRF of 2, followed by a gradual flux decrease, and ending with a steady-state flux. The initial decline of permeate flux can be attributed to quick blocking of the membrane pores due to retained particles. Further flux decline after pore blockage was due to the formation and growth of a cake layer on the membrane surface, which created an additional resistance to the permeate flow.

In the selected operating conditions, polyvinylidene fluoride (PVDF) HF membranes showed higher initial (75 L/m^2^h) and steady-state (43.5 L/m^2^h) permeate fluxes in comparison to polysulphone (PS) membranes (initial permeate flux of 27 L/m^2^h and steady-state permeate flux of 4.8 L/m^2^h). A similar behavior was also observed in the clarification of pomegranate juice with PVDF and PS HF membranes [27]. The PVDF membranes presented, in fact, higher permeate fluxes in comparison with PS membranes. de Carvalho et al. [28] evaluated the performance of different polymeric MF and UF membranes in the clarification of pineapple juice. In addition, in this case, the productivity of PVDF membranes was higher compared to that measured for PS membranes. The most relevant physico-chemical and nutritional properties of the aqueous extract (feed), the clarified extract (permeate) and the retentate stream coming from both UF treatments are reported in Table 1.

**Figure 2:**
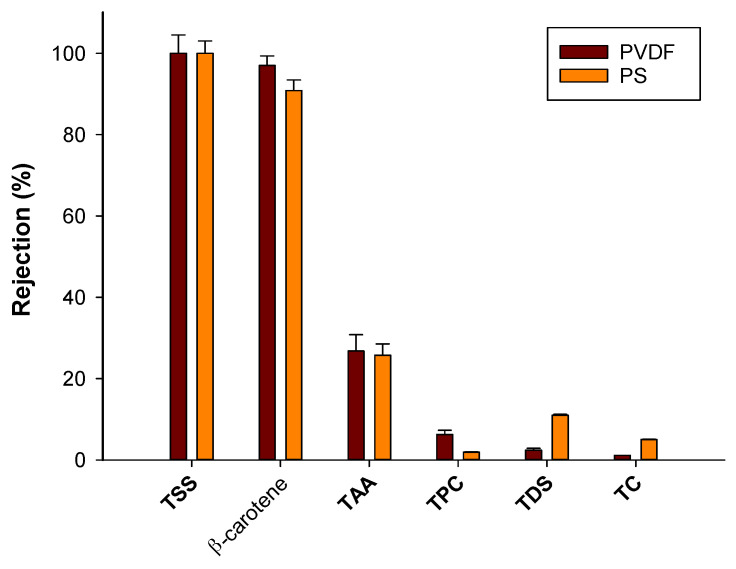
Rejection of hollow fiber UF membranes towards analyzed compounds (PS, polysulphone; PVDF, polyvinylidene fluoride; TSS, total suspended solids; TDS, total dissolved solids; TPC, total phenolic content; TAA, total antioxidant activity; TC, total carbohydrates).

The UF membranes produced a clarified extract free of suspended solids. On the other hand, most of the total dissolved solids (TDS) and total carbohydrates (TC) were recovered in the clarified extracts. The rejection of selected membranes towards these components was in the range of 1–11% (Figure 2).

As previously reported, the health benefits of Goji berries are mainly attributed to the presence of polyphenols and carotenoids [29]. Increased intake of these compounds, as demonstrated by different epidemiological studies, was associated with a reduction of different diseases including cardiovascular dysfunction, neurodegenerative diseases and different typologies of cancer [9]. The beneficial effects of polyphenols and carotenoids are mainly attributed to their capacity to counteract conditions of oxidative stress that are responsible for these diseases. Carotenoids and polyphenols have been demonstrated to have antioxidant properties in vitro, as they show scavenging activity against free radicals and singlet oxygen quenching abilities [30,31]. Analyses of polyphenols according to the Folin–Ciocalteau method revealed that these compounds were also very well preserved in the clarified fractions. Selected UF membranes exhibited a rejection towards polyphenols in the range 2–10% (Figure 2). However, a different trend was observed for β-carotene: the concentration of this component in the permeate streams was very low (0.26 ± 0.05 μg/mL and 0.098 ± 0.02 μg/mL for PS and PVDF membranes, respectively) in comparison to the untreated Goji extract (2.80 ± 0.5 μg/mL and 3.16 ± 0.1 μg/mL for PS and PVDF membranes, respectively). Therefore, both membranes retained β-carotene, yielding an uncolored permeate; accordingly, the observed rejection towards β-carotene was higher than 90%.

The high rejection level of β-carotene, with a MW (536.9 g/mol) smaller than the MWCO of the investigated membranes could be attributed to its bind to other larger molecules such as proteins and pectins [32]. In addition, β-carotene can be also attached to cell debris and lipids due to its hydrophobic character and rejection by membranes [33]. These results are in agreement with those reported by different Authors. Vaillant et al. [34] observed a total rejection of β-carotene during the clarification of melon juice with ceramic multichannel membranes (Membralox 1P19-40; Pall-Exekia, Bazet, France) having a pore diameter of 0.2 μm. A rejection higher than 90% towards lycopene (MW 536.9 g/mol) has been also reported by Paes et al. [35] in the treatment of papaya pulp (*Carica papaya* L.) with two different PS membranes, with a MWCO of 100 kDa and 50 kDa. Similarly, a PS 100 kDa membrane showed a rejection of about 98% against carotenoids from pequi (*Caryocar Brasiliense Camb.*) aqueous extract [36].

According to data reported in Table 1, the total antioxidant activity (TAA) of the UF feed is about 3.5 ± 0.5 mM and 2.8 ± 0.3 mM for PS and PVDF membranes, respectively. Clarified fractions contained a lower value of TAA (about 74% of TAA if compared with the feed solution). On the other hand, the rejection of UF membranes towards TAA was higher than 26%. This phenomenon could be attributed to the low concentration of β-carotene in these fractions, since this component together with polyphenols contributes to the antioxidant potential of Goji berry extracts.

### 2.2. Influence of Molecular Weight Cut-off and Transmembrane Pressure on the Performance of UF Membranes

**Figure 3:**
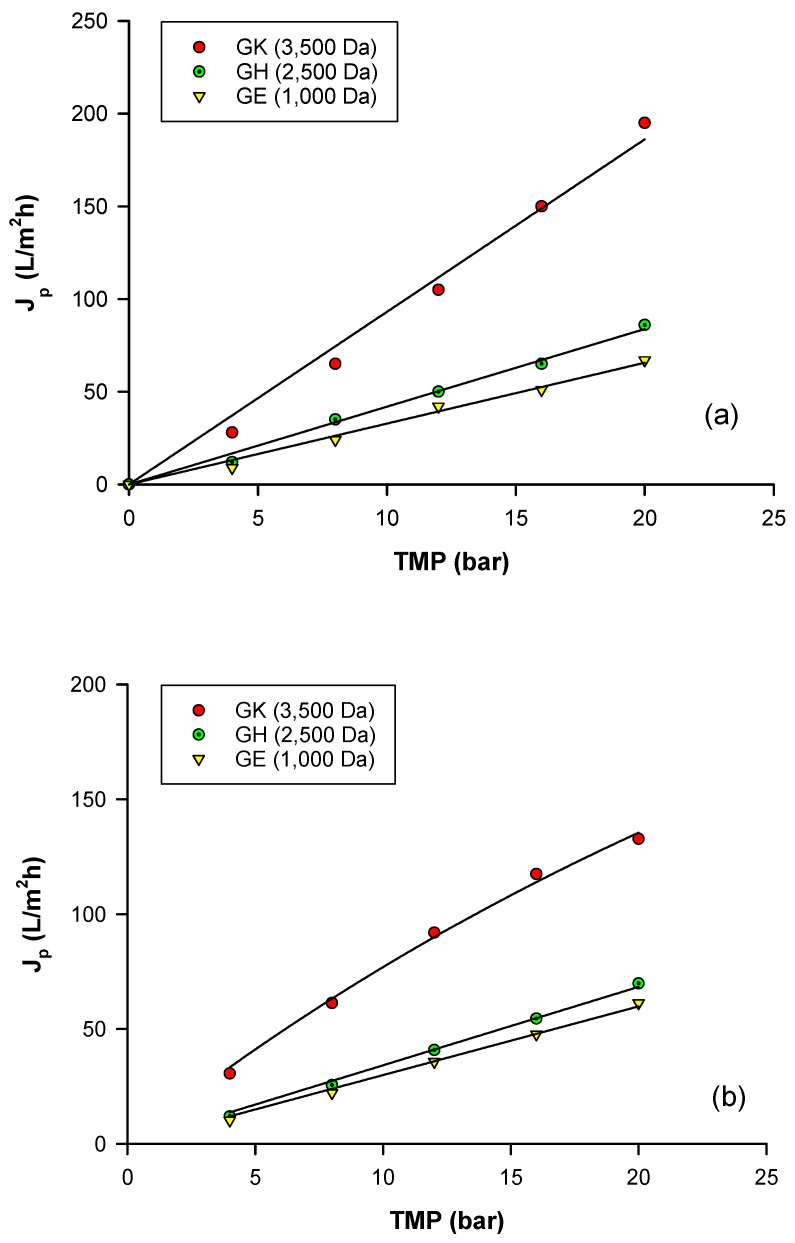
Permeate flux as a function of applied transmembrane pressure (TMP) for selected membranes with: (**a**) deionized water; (**b**) clarified Goji berries extract.

The clarified extract was fractionated by using three polymeric membranes in polyamide with a molecular weight cut-off (MWCO) in the range 1000–3500 Da. A first set of experiments was performed in order to evaluate the effect of MWCO and transmembrane pressure (TMP) on membrane productivity and selectivity towards phenolic compounds and sugars. The selected membranes were previously tested for their water permeability by measuring the water flux at different TMP values. As expected, an increase in the water flux was observed by increasing the MWCO, since larger pore sizes lead to higher permeate fluxes; indeed the highest permeability was measured for the GK membrane (W_p0_ 9.26 L/m^2^hbar) in comparison with the GH (W_p0_ 4.19 L/m^2^hbar) and GE (W_p0_ 3.28 L/m^2^hbar) membranes (Figure 3a). The effect of TMP on the permeate flux in the treatment of the clarified extract according to a total recycle configuration is shown in Figure 3b. Experimental results indicated a quite linear increase in the permeate flux by increasing the applied TMP due to minimal concentration polarization and fouling phenomena. The absence of a limiting flux can be attributed to the removal of macromolecules, colloidal species and suspended solids in the preliminary clarification step of the Goji extract by UF. As is well known, these compounds tend to form a deposited foulant layer over the membrane surface, adding reversible membrane resistance to the total membrane resistance [37].

These results are consistent with other studies performed with different fruit juices and vegetable solutions. A linear trend between TMP and permeate flux was reported in the treatment of clarified pomegranate juice using a GK membrane [38]. Similarly, Diaz-Reinoso et al. [39] observed a linear increase of permeate flux with pressure in the processing of aqueous extracts from distilled fermented grape pomace using a GE membrane.

As reported in Figure 3b, the permeate flux increased by increasing the MWCO as follows: the GK membrane, exhibited higher permeate fluxes when compared with other investigated membranes. On the other hand, the GE membrane showed the lowest permeation flux. This behavior can be attributed to an increasing resistance to the permeate flux exerted by membranes with lower MWCO [40]. A similar trend was also observed in the separation and purification of ellagitannins from clarified blackberry juice with thin film composite membranes from GE Osmonics (GK and GH) [41].

The water permeability of the selected membranes was measured before and after the treatment of clarified Goji extract in order to evaluate the degree of membrane fouling. The obtained results, together with measurements of fouling index (FI) and cleaning efficiency (CE), are reported in Table 2. The lowest FI value was measured for the GH membrane (4.06%), followed by the GK membrane (6.9%), while the highest value was detected for the GE membrane (43.9). The CE was higher than 94% for all investigated membranes, according to the following order: GH > GK > GE.

**Figure 4:**
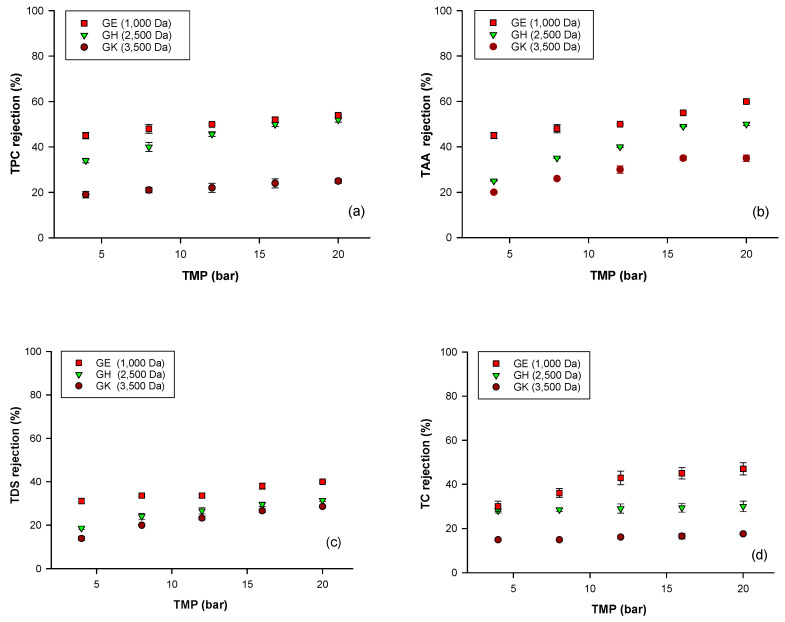
Influence of TMP and MWCO of selected membranes on the rejection of analyzed compounds. (**a**) TPC, total phenolic content; (**b**) TAA, total antioxidant activity; (**c**) TDS, total dissolved solids; (**d**) TC, total carbohydrates.

The rejection of the selected membranes towards analyzed compounds as a function of the applied TMP is reported in Figure 4. For each membrane, the rejection towards analyzed compounds increased by increasing the applied pressure, in the range of TMP investigated (4–20 bar). This behavior could be attributed to a higher polarization concentration and fouling phenomena when TMP is increased, leading to the formation of a cake layer on the membrane surface and to an increase of the rejection coefficient [24,42]. Secondly, the rejection increased by decreasing the MWCO of the UF membranes. Therefore, the rejection coefficients were higher for the GE membrane.

**Figure 5:**
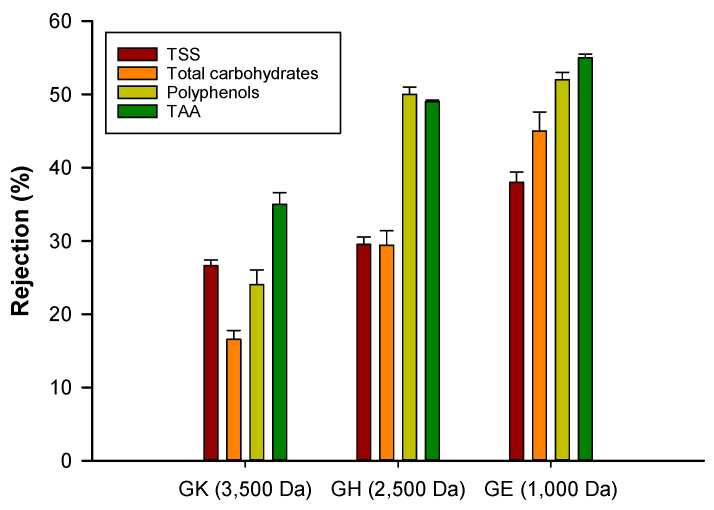
Rejection of GK, GH and GE membranes towards analyzed compounds at a TMP of 16 bar.

These results are in agreement with those of several studies related to the treatment of different vegetable extracts that also reported high rejection of solutes by increasing the applied TMP and decreasing the MWCO of UF and NF membranes [24,39,41,43]. It is expected that a membrane with small pore size retains a large amount of solutes; on the other hand, if the pore size is larger, the molecules pass more easily through the membrane and can be recovered in the permeate fraction. In particular, the GE membrane showed at each applied pressure, the highest rejection towards polyphenols, TAA, TSS and total carbohydrates (Figure 4a–d). On the other hand, the GK membrane allowed us to recover almost all of the analyzed compounds in the permeate stream due to its lower rejection. Different results have been obtained with the GH membrane. In particular, at a selected TMP of 16 bar, this membrane showed a rejection towards TDS and total carbohydrates lower than 30%, while the observed rejection towards polyphenols and TAA was about 50%, indicating the best separation factor for this membrane for antioxidant compounds (mainly polyphenols) from the clarified berries extract (Figure 5). Therefore, this membrane was selected to perform further experiments to improve the separation of sugar compounds from phenolics.

### 2.3. Experiments in Diafiltration and Concentration Mode with GH Membrane

A focus on the obtained results in terms of selectivity, fouling index and cleaning efficiency allowed us to select the GH membrane for other experiments, both in diafiltration (in order to improve the purification of polyphenols) and batch concentration mode (in order to increase the concentration of polyphenols in the retentate stream). This set of experiments was performed by using a GH membrane in a spiral-wound configuration.

**Figure 6:**
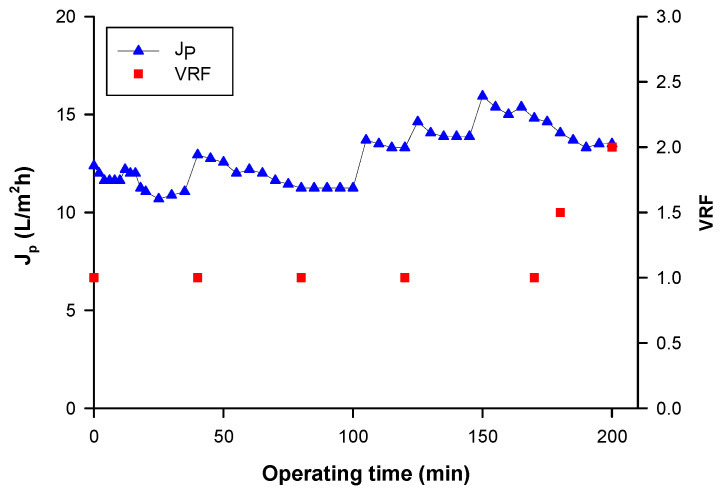
Treatment of clarified Goji berries extract with GH membrane. Time course of the permeate flux and VRF (Operating conditions: diafiltration/batch concentration; TMP, 16 bar, T, 30 ± 1 °C).

Figure 6 shows the evolution of permeate flux as a function of VRF in the treatment of the clarified Goji extract according to the diafiltration/concentration operating mode. The re-dilution with water of the feed during initial diafiltration experiments showed a positive impact on permeate flux. In particular, as soon as the feed was filled up with water, an increase in flux was observed up to its initial value (13 L/m^2^h) or in some cases higher (16 L/m^2^h), indicating that the addition of water reduces the osmotic pressure of the feed and a higher driving force filtration is obtained [44]. The diafiltration process was applied until a diafiltration volume of 3 was reached; then the retentate was processed in batch concentration mode up to a VRF of 2. In these conditions, the initial permeate flux of 15 L/m^2^h was reduced up to 13.50 L/m^2^h when the final VRF was achieved.

The chemical composition of clarified extract and retentate fractions coming from the diafiltration/concentration process is reported in Table 3. According to the obtained results, the concentration of total dissolved solids and total carbohydrates was strongly reduced after the diafiltration process with a decrease of these compounds in the retentate fraction of about 77% and 64%, respectively. On the other hand, the content of polyphenols and TAA remained similar to the initial value with a loss in the retentate lower than 4%. The final concentration process allowed us to increase the concentration of polyphenols and TAA in the retentate side of about 80% and 60%, respectively, if compared with the clarified extract. Consequently, the efficiency of the GH membrane in the purification of polyphenols from the Goji berries extract is enhanced after the diafiltration/concentration process due to the different selectivity of the membrane towards these compounds: adding water increases the purification factor of polyphenols in the retentate, while the concentration of sugars increases in the permeate (diafiltrate). The final concentration step allowed us to obtain a fraction enriched in polyphenols with high antioxidant capacity and depleted in sugar compounds. The latter could be of interest in the development of functional foods or in the formulation of pharmaceutical and cosmestic products considering the health and anti-aging properties of Goji berries. The permeate of the diafiltration/concentration step, enriched in sugars, could be of interest in the food industry.

## 3. Material and Methods

### 3.1. Aqueous Extract from Goji Berries

Fresh Goji berries of Calabria origin were provided by Favella (Corigliano Calabro, Cosenza, Italy). Berries were washed in cold tap water in order to remove foreign materials. Then the fruits were squeezed by using an electric juicer (Aristalco, S.r.l., Treviso, Italy). The obtained puree was diluted with distilled water (liquid-to-solid ratio 5 mL/g) and thermostated in a water bath at 80 °C for 30 min. The aqueous extract was filtered through a nylon cloth to remove solid particles and seeds. The extract was then stored at −17 °C and defrosted to room temperature before use.

### 3.2. Clarification of Aqueous Extract: Equipment and Procedures

The aqueous extract was clarified by using a laboratory bench plant consisting of a feed tank with a capacity of 4 L, a magnetic drive gear pump (Micropump GC-M25 JF5 SA), two pressure gauges located at the inlet (P_in_) and outlet (P_out_) of the membrane module, a pressure control valve and a permeate tank. A cooling coil fed with tap water was used to control the feed temperature. Two hollow fiber membrane modules made in PS and PVDF were used to clarify the aqueous extract. Their characteristics are reported in Table 4.

Experiments were performed according to the batch concentration configuration (collecting separately the permeate stream and recycling continuously the retentate in the feed tank) in selected operating conditions of pressure (0.8 bar), temperature (21 ± 1°C) and feed flowrate (280 L/h) up to a VRF of 5.

The VRF is defined as the ratio between the initial feed volume and the final retentate volume, according to the following Equation (1):

(1) $$VRF=\frac{V_{f}}{V_{r}}=1+\frac{V_{p}}{V_{r}}$$

where *V_f_*, *V_p_*, and *V_r_* are the volume of feed, permeate, and retentate, respectively.

A digital balance was used to evaluate the permeate flux by measuring the volume of permeate collected in a certain time through the membrane surface area. Permeate flux was expressed as L/m^2^h.

The water permeability of each membrane module was determined by the slope of the straight lines obtained by plotting the water flux values in selected operating conditions versus the applied transmembrane pressure (TMP). After the clarification step, PS and PVDF membrane modules were cleaned in two and three steps, respectively. The first cleaning step was performed by recirculating distilled water for 20 min through the selected membrane modules. In the second step both membrane modules were submitted to a cleaning procedure using a 1% *w/w* enzymatic solution (Ultrasil 53, Henkel KGaA, Dusseldorf, Germany), which was recycled through the membrane module at 40 °C for 60 min followed by rinsing with tap water for 20 min.

An additional cleaning step was performed for the PVDF membrane module by using a 2.8% sodium hypochlorite solution at 40 °C for 60 min, followed by rinsing with tap water for 20 min. The cleaning protocol allowed us to recover about 85% of the initial water permeability of both UF membranes.

### 3.3. Treatment of Clarified Extract with Tight UF Membranes: Equipment and Procedures

#### 3.3.1. Screening Tests

Preliminary fractionation/concentration experiments were performed by using three polymeric membranes in flat sheet configuration with a MWCO in the range 1000–3500 Da (namely GE, GH and GK) manufactured by GE Osmonics and purchased from Sepra S.r.l. (Cesano Maderno, Italy). Membrane codes and technical specifications are reported in Table 4.

UF experiments were performed in total recycle mode (the feed composition was kept constant by recycling both permeate and retentate to the feed tank) in order to study the membrane performance in terms of productivity and selectivity towards target compounds, at different TMP values. At the end of the experimental runs, each membrane was cleaned by recirculation of tap water for 20 min; then the fouled membranes were cleaned with an enzymatic solution (1% *w/w* Ultrasil 50) at 40 °C for 60 min.

The fouling index (FI), expressed as a percentage drop in the water permeability, was estimated by measuring the water permeability before and after the treatment of the aqueous extract, according to the following Equation (2):

(2) $$FI=\left(1-\frac{W_{p1}}{W_{p0}}\right)\times 100$$

where *W_p_*_1_ is the water permeability after the treatment of clarified extract and *W_p_*_0_ the initial water permeability.

The cleaning efficiency (CE) was evaluated by using the water flux recovery method, according to the following Equation (3):

(3) $$CE=\left(\frac{W_{p2}}{W_{p0}}\right)\times 100$$

where *W_p_*_2_ is the water permeability measured after the enzymatic cleaning.

#### 3.3.2. Experiments with the GH Membrane in Diafiltration and Concentration Mode

Based on the preliminary results, the GH membrane with the best overall performance, was selected for further experiments in diafiltration and batch concentration mode in order to improve the concentration and purification of polyphenols. This set of experiments was performed by using a pilot unit (DeltaE s.r.l., Cosenza, Italy) equipped with a stainless steel housing able to accommodate a spiral-wound membrane module featuring an effective membrane area of 0.32 m^2^. The equipment consists of a stainless steel feed tank, a digital flowmeter (SM6000, ifm electronic gmbh, Essen, Germany), a high pressure pump (Cat Pumps, Milano, Italy, Model 3CP1221), two pressure gauges (Wika Instrument, Lawrenceville, GA, USA) and a control panel. The feed temperature was adjusted by circulating tap water in the two-layered feed tank. The adjustment of operating pressure and feed flow rate was done by simultaneous pump rotation control through a frequency inverter and a needle valve located after the membrane module.

During diafiltration experiments a quantity of solvent (water) was added at the same rate as the permeate was recovered. Experiments were performed in selected operating conditions (TMP, 16 bar; T, 30 °C) until to a diafiltration volume of 3 and a VRF of 2, respectively, were reached. 

The dialfiltration volume (DV) is defined as the ratio of the solvent volume added per volume of feed solution [45]. 

The observed rejection (*R*, %) values were calculated with the following Equation (4):

(4) $$R=\left(1-\frac{C_{p}}{C_{r}}\right)\times 100$$

where *C_p_*, *C_f_* and *C_r_* are the concentrations of a specific compound in the permeate, feed and retentate, respectively.

### 3.4. Chemical Analysis and Determinations

#### 3.4.1. Total Dissolved Solids (TDS) and Total Suspended Solids (TSS)

Total dissolved solids (TDS) measurements were carried out by using a hand refractometer (Atago Co., Ltd., Tokyo, Japan) with scale range of 0–32° Brix. Total suspended solids (TSS) were determined in relation to total juice (*w*/*w*, %) by centrifuging at 2000 rpm for 20 min with 15 mL of pre-weighted sample; the content of settled solids was recorded after removing the supernatant and after drying at 70 °C under vacuum for 48 h.

#### 3.4.2. β-Carotene

The concentration of β-carotene was determined according to a spectrophotometric method [46] by using the following Equation (5):

(5) $$C_{\beta -carotene}=4.624\;\times A_{450}-3.091\times A_{503}$$

where *C* is the concentration of carotenoids expressed in μg/mL, and A450 and A503 represent the absorbance at 450 nm and 503 nm, respectively.

#### 3.4.3. Total Phenolic Content

Total phenolic content (TPC) was measured colorimetrically by the Folin–Ciocalteau method [47]. The sample (0.2 mL) was mixed with 1 mL of 10% (*w*/*v*) Folin–Ciocalteu reagent. After 5 min, 2.5 mL of sodium carbonate solution (75 g/L) were added and mixed. After 30 min of incubation at room temperature the absorbance of the resulting solution was measured at 765 nm by using a UV-visible spectrophotometer (Shimadzu UV-160A, Kyoto, Japan). Gallic acid solutions in the range of 10–100 ppm were used as a calibration standard (Figure S1) and results were expressed as mg of gallic acid equivalents per liter (mg GAE/L), based on standard curve.

#### 3.4.4. In Vitro Antioxidant Activity

The total antioxidant activity (TAA) was assessed by the 2,2-azino-bis (ethylbenzothiazoline-6-sulfonic acid) (ABTS) assay by monitoring the reduction of the radical cation as the percentage inhibition of absorbance at 734 nm [48,49].

The ABTS radical cation was produced by reaction of 10 mL of ABTS stock solution with 100 mL of 70 mM potassium persulphate (K_2_S_2_O_8_) (ABTS: K_2_S_2_O_8_ = 1:0.35 M ratio). The mixture was stored in the dark at room temperature for 12–14 h, before use. After addition of 10 mL of sample (antioxidant) to 10 mL of ABTS work solution, the absorbance at 734 nm was measured every min for a total of 6 min. 6-hydroxy-2,5,7,8-tetramethylchroman-2-carboxylic acid (Trolox) solutions in the range of 0.5–1.5 mM were used for calibration (Figure S2) and results were expressed in terms of mM Trolox equivalents.

#### 3.4.5. Total Carbohydrates

Total carbohydrates (TC) were measured by using the phenol-sulfuric acid method [50]. A sample aliquot (0.2 mL) of a carbohydrate solution is mixed with 1 mL of 5% aqueous solution of phenol in a test tube. Then, 5 mL of concentrated sulfuric acid is added rapidly to the mixture. After allowing the test tubes to stand for 10 min, they are vortexed for 30s and placed for 30 min in a water bath at room temperature for color development. The absorbance was measured at 420 nm by using an UV-visible spectrophotometer (Shimadzu UV-160 A, Kyoto, Japan). Glucose solutions in the range of 0.02–0.1 g/L were used for calibration (Figure S3). A dose response linear regression was generated by using the glucose standard absorbance and results were expressed as g glucose/L.

## 4. Conclusions

Research for new functional ingredients from natural sources is one of the most important challenges in the food and pharmaceutical industries.

In this work the integration of ultrafiltration-based membranes with different MWCO for the concentration of β-carotene and polyphenols from Goji berries aqueous extracts was investigated. Preliminary clarification experiments with hollow fiber membranes allowed us to produce a clarified aqueous extract with chemical and nutritional properties comparable to those of fresh aqueous extracts, except for the absence of suspended solids and carotenoids. The permeate of both processes is a clear solution enriched in polyphenols and sugars with high antioxidant capacity. The clarified extract was fractionated with polymeric membranes with different molecular weight cut-offs. Among the selected membranes, the GH membrane exhibited a higher rejection towards phenolic compounds (higher than 50%) and a lower rejection towards total soluble solids and carbohydrates (lower than 30%). The inclusion of a diafiltration process allowed us to improve the concentration of sugar compounds in the permeate stream and to increase the purification of polyphenols in the retentate fraction. According to the obtained results, the investigated process for the treatment of Goji berries aqueous extracts produced three different valuable streams:

a retentate fraction from the clarification process that could be considered a new source of β-carotene for different food applications (i.e., as natural, nontoxic food colorants or food supplements);
a retentate fraction from the diafiltration/concentration process with a GH membrane enriched in polyphenols with high antioxidant capacity of interest in the food, pharmaceutical and cosmetic industries;
a permeate fraction from the diafiltration/concentration process with a GH membrane enriched in sugars of interest for applications in the food industry.

## Figures and Tables

**Table 1:** Chemical composition of Goji berry extract clarified by hollow fiber membranes.

Parameters	PS Membranes			PVDF Membranes		
	Feed	Permeate	Retentate	Feed	Permeate	Retentate
TSS (%)	1.6 ± 1.1	n.d.	5.8 ± 0.2	1.6 ± 0.2	n.d.	5.8 ± 0.1
TDS (° Brix)	4.5 ± 0.4	4.0 ± 0.2	4.5 ± 1.2	4.2 ± 0.1	4.1 ± 0.2	4.2 ± 0.05
β-carotene (μg/mL)	2.80 ± 0.5	0.26 ± 0.05	4.74 ± 0.25	3.16 ± 0.1	0.098 ± 0.02	4.34 ± 0.35
TPC (mg GAE/L)	520.5 ± 2.6	510.0 ± 6.0	540.0 ± 8.0	448.5 ± 2.6	420 ± 6.0	480 ± 6.0
TAA (mM Trolox)	3.5 ± 0.5	2.6 ± 0.2	3.4 ± 0.12	2.8 ± 0.3	2.05 ± 0.25	2.6 ± 0.4
TC (g glucose/L)	15.1 ± 2.3	14.35 ± 1.5	18.2 ± 2.3	17.7 ± 3.2	17.5 ± 2.6	18.67 ± 2.4

PS, polysulphone; PVDF, polyvinylidene fluoride; TSS, total suspended solids; TDS, total dissolved solids; TPC, total phenolic content; TAA, total antioxidant activity; TC, total carbohydrates.

**Table 2:** Water permeabilities, fouling index and cleaning efficiency of selected UF membranes.

	Membrane Type		
	GK	GH	GE
W_p0_ (L/m^2^hbar)	9.26	4.19	3.28
W_p1_ (L/m^2^hbar)	8.62	4.08	1.84
W_p2_ (L/m^2^hbar)	9.104	2.62	3.1
FI (%)	6.9	4.06	43.9
CE (%)	98.27	100	94.5

W_p0_, initial water permeability; W_p1_, water permeability after treatment with Goji berries aqueous extract; W_p2_, water permeability after enzymatic cleaning; FI, fouling index; CE, cleaning efficiency.

**Table 3:** Chemical composition of the clarified Goji berries extract before and after the diafiltration/concentration process with the GH membrane.

Parameters	Sample		
	Feed	Retentate at DV 3	Retentate at VRF 2
TDS (° Brix)	3.1 ± 0.4	0.7 ± 0.2	0.9 ± 0.5
TC (g glucose/L)	17.1 ± 0.4	6.2 ± 0.2	8.4 ± 0.5
TPC (mg GAE/L)	450 ± 1.3	432.4 ± 6.2	812.4 ± 10.2
TAA (mM Trolox)	3.0 ± 0.5	2.9 ± 0.6	4.8 ± 0.9

TDS, total dissolved solids; TC, total carbohydrates; TPC, total phenolic content; TAA, total antioxidant activity; DV, diafiltration volume; VRF, volume reduction factor.

**Table 4:** Characteristics of selected membranes.

Membrane Type	HFS	DCQ II-006 C-PS100	GE	GH	GK
Manufacturer	Toray, Tokyo, Japan	China Blue Star Membrane Technology Co. Ltd., Beijing, China	GE Osmonics, Minnetonka, MN, USA	GE Osmonics, Minnetonka, MN, USA	GE Osmonics, Minnetonka, MN, USA
Membrane material	PVDF	PS	PA-TFC	PA-TFC	PA-TFC
Configuration	Hollow fiber	Hollow fiber	Flat-sheet	Flat-sheet/Spiral-wound	Flat-sheet
Nominal MWCO(Da)	-	100,000	1000	2500	3500
Pore size (m)	0.02	-	-	-	-
pH operating range	2–12	2–13	2–10	2–10	2–10
Max. operating temperature (°C)	40	50	50	50	50
Max. operating pressure (bar)	2	1.5	27.6	27.6	27.6
Membrane surface area (m^2^)	0.4	0.16	0.0035	0.0035/0.32	0.0035

MWCO, molecular weight cut-off; PVDF, polyvinylidene fluoride; PS, polysulphone; PA, Polyamide; TFC, thin film composite.

## Supplementary Materials

The following are available online. Calibration curves for total phenol content, total antioxidant activity and total carbohydrates are supplied as Figures S1–S3, respectively.
