# Supplementary material for: Coupling Ultrafiltration-Based Processes to Concentrate Phenolic Compounds from Aqueous Goji Berry Extracts
Source: Molecules. 2020 Aug 18;25(16):3761. doi: 10.3390/molecules25163761 (PMC7547376; doi:10.3390/molecules25163761)
Supplement: Supplementary file 1 [file molecules-25-03761-s001.pdf]

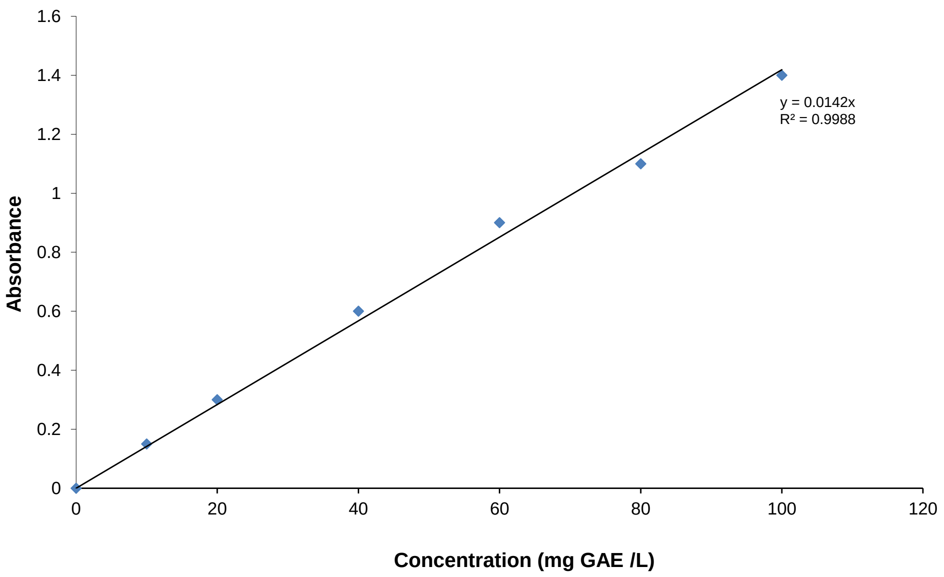


**Figure S1.** Gallic acid standard curve for total phenolic content.

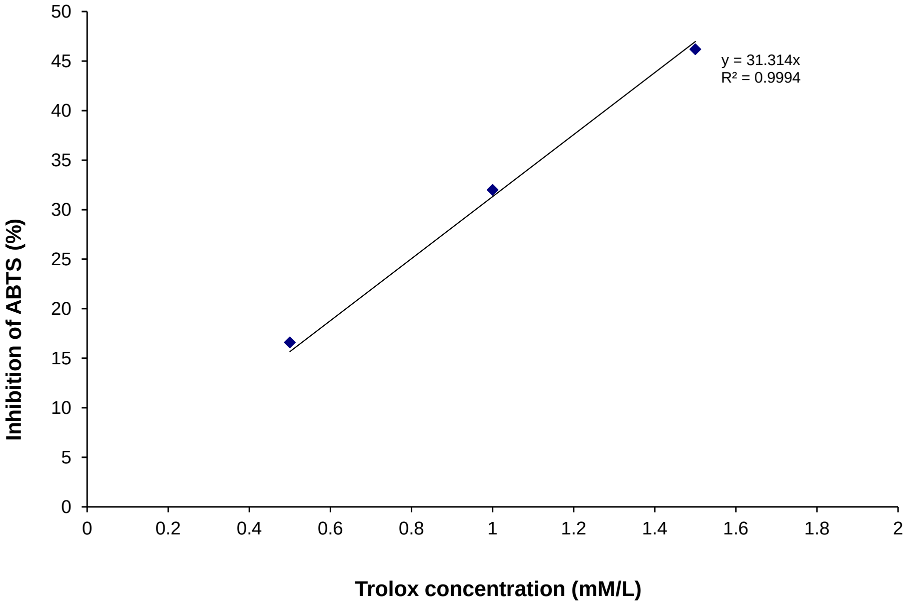


**Figure S2.** Calibration curve of ABTS inhibition by Trolox standards.

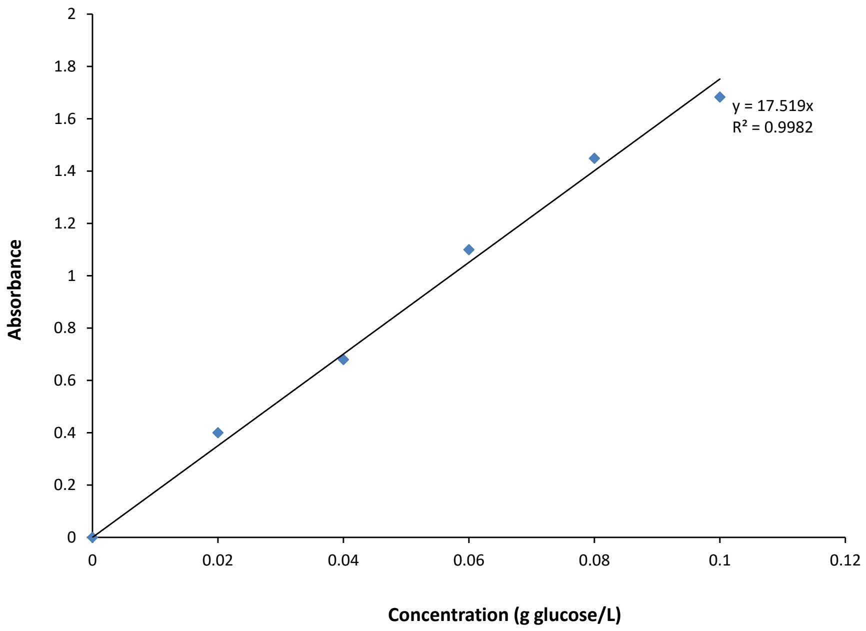


**Figure S3.** Standard curve of total carbohydrate content.
